# Successful Midfoot Reconstruction in Müller-Weiss Disease Using a Reverse Fibular Flap: A Case Report and Literature Review

**DOI:** 10.7759/cureus.68423

**Published:** 2024-09-02

**Authors:** Julia Slater, Neil McLean, David Townshend, Jonathan Powell

**Affiliations:** 1 General Surgery, Newcastle Hospitals NHS Trust, Newcastle, GBR; 2 Plastic Surgery, University of Adelaide, Adelaide, AUS; 3 Orthopedic Surgery, Northumbria NHS Trust, Newcastle, GBR; 4 Plastic Surgery, South Tees Hospitals NHS Trust, Middlesbrough, GBR

**Keywords:** orthopedic surgery, müller-weiss disease, plastic surgery, foot reconstruction, reverse fibular flap

## Abstract

Müller-Weiss disease (MWD) is a poorly understood orthopedic condition first described in 1927 that causes chronic pain across the midfoot and hindfoot. The etiology is uncertain but includes navicular dysplasia, osteochondritis, and trauma. The initial management is conservative, aiming to reduce the patient's symptoms, and includes analgesia, footwear, and activity modification. Surgical interventions such as joint fusion are considered when conservative measures fail, but there is little recorded for treatment beyond this.

This case outlines the difficult management of a 52-year-old female patient with a long history of MWD. She had no history of previous trauma or neurological problems. A talonavicular fusion failed to unite, resulting in significant necrosis of the lateral navicular and navicular-cuneiform arthritis. We describe the novel use of a reverse vascularized pedicled fibular flap and extended midfoot fusion to manage the navicular bone defect. At six-year follow-up, the patient remains virtually pain-free and has returned to work with radiographs confirming good incorporation of the bone graft.

We understand this to be the first documented use of a reverse vascularized fibular bone graft for recalcitrant MWD. Given the excellent clinical outcome in this case, surgeons should consider this combined ortho-plastics approach in the management of complex non-union with a bone defect in the midfoot.

## Introduction

In 1927, Walther Müller described a chronic condition of the midfoot characterized by progressive pain and deformity which he attributed to long-term compression of the navicular bone. The same year, radiologist Konrad Weiss identified similar findings among his own patients. It was at this time that the condition was labeled Müller-Weiss disease (MWD) [1,2].

The navicular begins to ossify between the ages of 18 and 36 months and articulates with the talus, cuboid, and cuneiform bones [3,4]. The pathogenesis and incidence of MWD remain unclear, which may reflect the limited knowledge among clinicians and its potential misdiagnosis. MWD was originally thought to be an osteonecrotic process of the navicular in adults and draws similarities to Köhler disease, avascular necrosis of the tarsal navicular in children [4,5]. However, Doyle et al. concluded the two are not related, as Köhler disease demonstrates a complete clinical and radiological resolution in childhood [6]. Maceira and Rochera suggested the condition results from delayed ossification of the navicular bone; in this weakened state, there may be an increased risk of fragmentation [3,5]. Other possible etiologies include dysplasia due to increased lateral loading of the cartilaginous navicular, osteochondritis, and trauma [5]. Maceira and Rochera also suggested a classification of MWD, graded from stages 1 to 5, based on radiological findings including angulation of the Meary-Tomeno line and loss of the longitudinal arch [5]. The epidemiology of MWD is unknown; theories linking increased prevalence with areas of extreme poverty or political unrest have been disproven [6].

There is currently no gold standard management for MWD, with treatment aimed at reducing the individual’s symptoms [7]. Patients typically present with mid and hindfoot pain and it is often related to secondary osteoarthritis [8]. Patients present between the ages of 40 and 60 and women are more commonly affected than men [1,3]. Radiographs frequently show a “comma”-shaped deformity due to the collapse of the lateral portion of the navicular [3,5,8]. A short first metatarsal with secondary arthritis of the navicular and its surrounding joints is also common [8]. The condition may be bilateral and pathological fractures do occur [8]. Non-operative management can be successful in the majority of cases and involves analgesia as well as orthotics [3]. If non-operative management fails, then surgical intervention may be warranted. This should be focused on fusing symptomatic joints and restoring the medial longitudinal arch [3,7]. Doyle et al. suggested that resistant MWD can be successfully managed by triple fusion, with or without the inclusion of the ﻿naviculocuneiform joint [6].

This case highlights the difficulty in treating a patient with MWD and the novel use of a reverse flow fibular flap (FF) to fuse the bones of the midfoot and restore a normal pain-free gait.

## Case presentation

**Figure 1:**
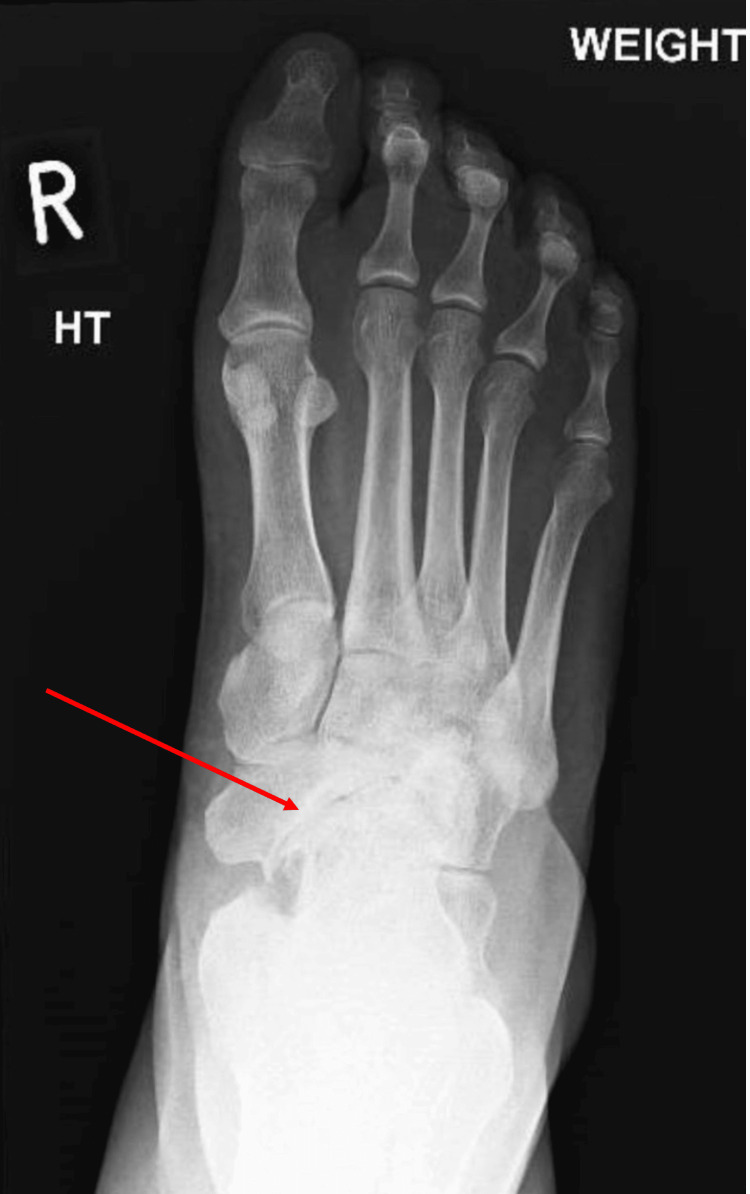
Antero-posterior radiograph of the right foot, arrow demonstrates secondary arthritic changes

**Figure 2:**
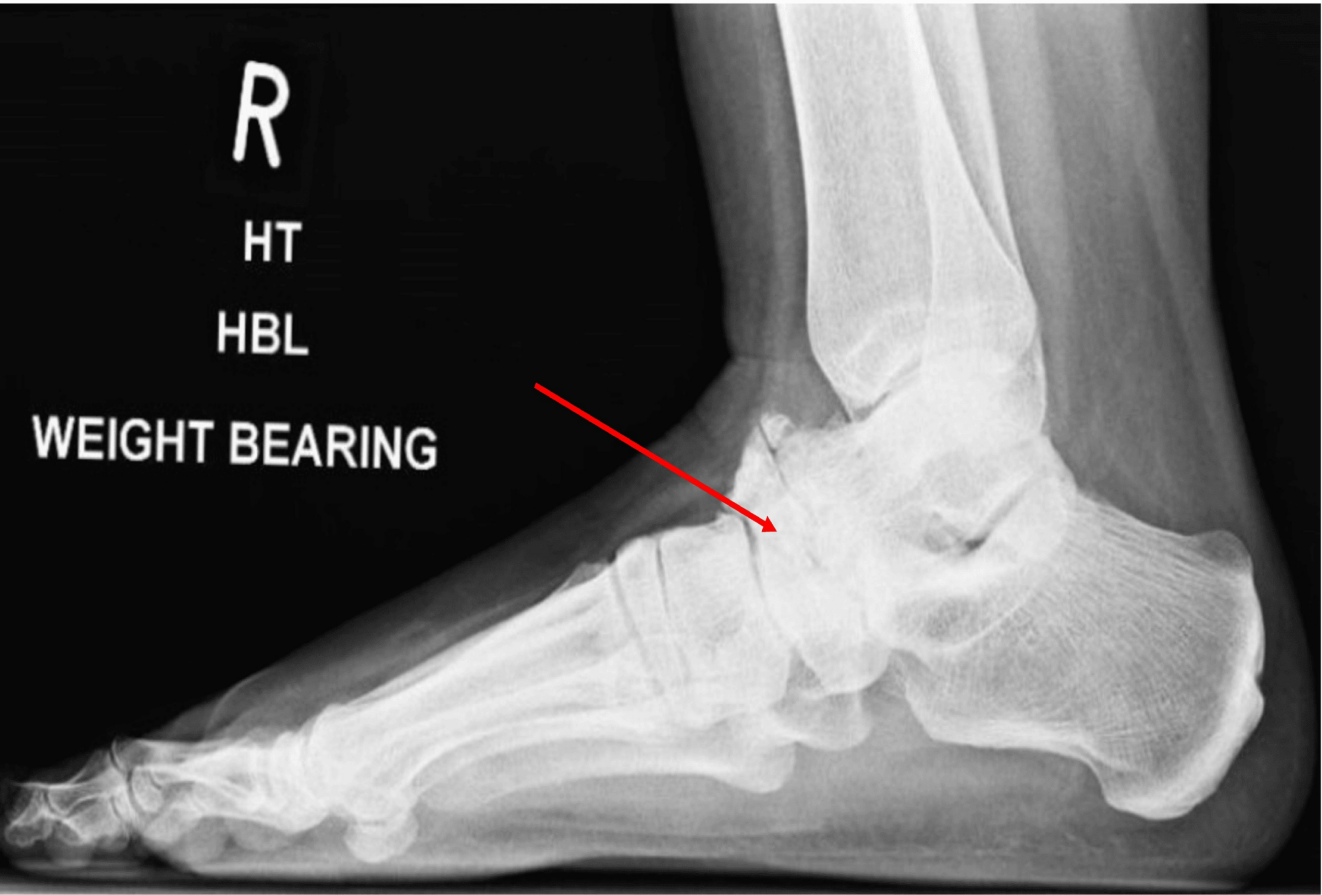
Lateral radiograph of the right foot, arrow demonstrates navicular collapse and arthritic changes

In 2011, a 52-year-old female presented with worsening of her longstanding severe bilateral foot pain. Initial radiographs are presented in Figures 1, 2 as anteroposterior and lateral views of the right ankle which demonstrate collapse of the navicular and secondary osteoarthritic changes. She was fitted with an orthosis; however, the disease process progressed. In 2014 the patient underwent an unsuccessful left talonavicular fusion with an iliac bone graft which was repeated in 2015 with a successful outcome.

The right foot continued to deteriorate clinically, with worsening pain on everyday tasks and the patient was referred to the pain team and physiotherapy services for pain management. Computerized tomography (CT) imaging demonstrated a severely arthritic right talonavicular joint. In 2016 a right talonavicular fusion with a uni-cortical iliac crest bone graft, cancellous bone chips, and a CLAW 2 rigid plate fixation was carried out. This procedure proved unsuccessful, with loosening of the metalware and fracture of a screw. The patient continued to have severe pain and required a walking stick to mobilize. Sequential CT scans and radiographs between 2016 and 2018 revealed partial bony bridging with poor incorporation of the iliac bone graft and evidence of navicular-cuneiform osteoarthritis as well as breakage of the CLAW 2 plate. A subsequent course of Exogen Bone Stimulator therapy was unsuccessful. Further imaging revealed degeneration of the right talonavicular joint with minor degeneration of the calcaneocuboid joint.

Following discussion at the complex foot and ankle multidisciplinary team (MDT) meeting a vascularized bone graft was suggested. A review of the available literature identified two papers discussing navicular non-unions and one reviewing bone grafting in the foot and ankle in general. Fishman et al. reported a combination of cuneiform and cuboid pedicle grafts in eight patients with a 75% union rate [9]. Holm et al. described the use of a vascularized medial femoral condyle flap to successfully fuse the talonavicular and naviculocuneiform joints in a 48-year-old female [10]. Haddock et al. reviewed 12 patients who underwent vascularized bone grafting to the foot and ankle between 2006 and 2012. One of these patients (a 52-year-old female) suffered from navicular avascular necrosis and underwent a vascularized medial femoral condyle graft which successfully incorporated, leading them to conclude that the use of the medial femoral condyle as a vascularized graft in the small bones of the foot and ankle was an option [11].

**Figure 3:**
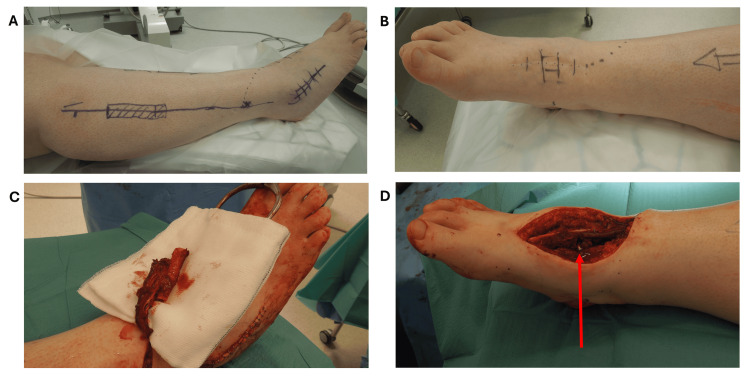
A) Pre-operative markings of 10 cm fibular bone graft. B) Pre-operative marking over navicular recipient site. C) Intra-operative view of transfer of vascularized fibular graft. D) Intra-operative view of fibular graft, marked by an arrow, in position in midfoot

**Figure 4:**
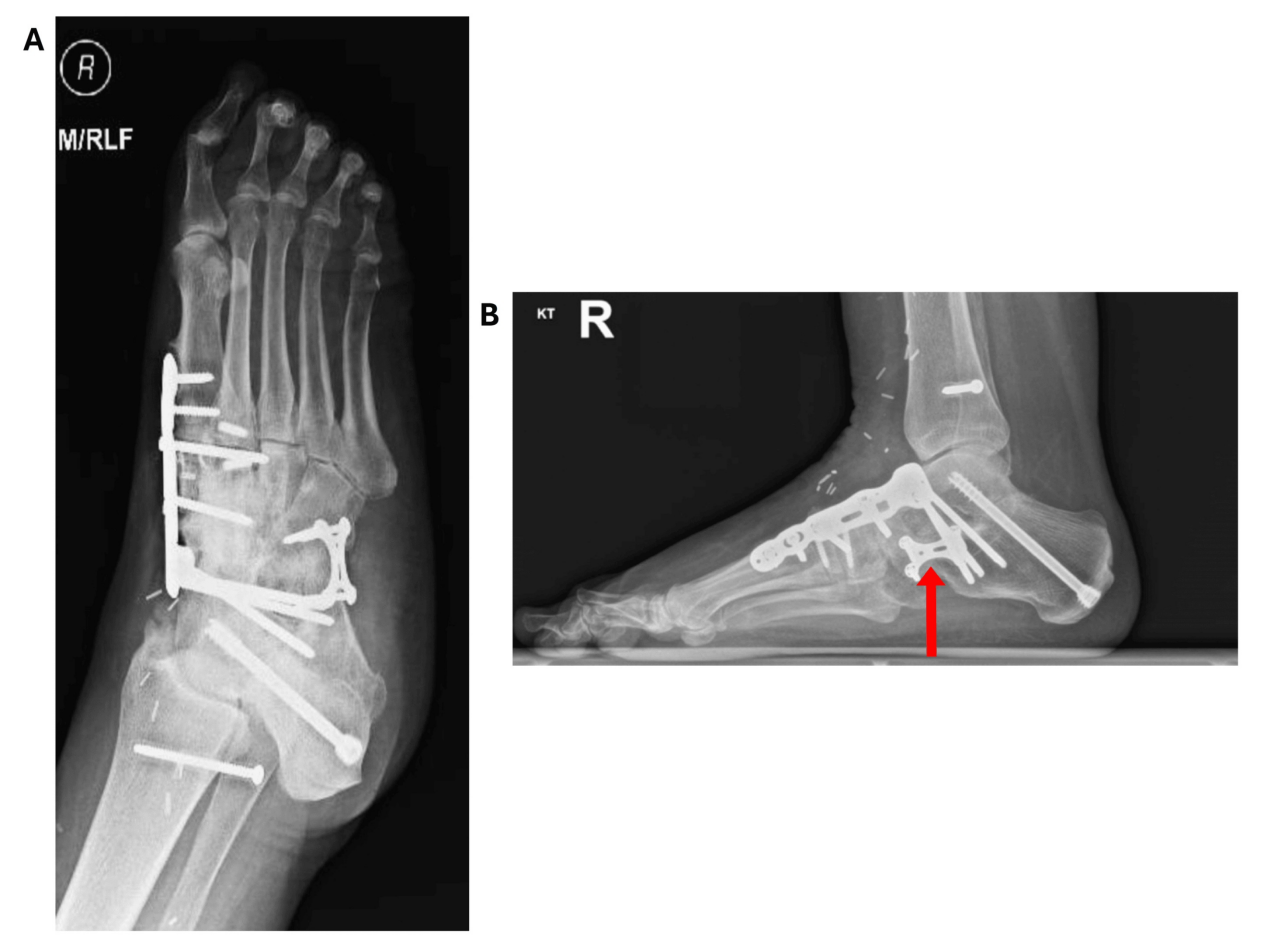
A) Oblique and B) lateral radiographs taken at two-year follow-up demonstrating successful incorporation of the bone graft and intact metalware, marked by an arrow

In 2018 a decision was made to carry out a reverse-flow vascularized fibular bone graft to allow for a revision midfoot triple fusion. Later that year the patient underwent a revision fusion of the right talonavicular-cuneiform joint with a reverse-flow fibular osseous flap of 10 cm in length and a vascular pedicle of the peroneal vessels of 8 cm in length, which fused the subtalar and calcaneo-cuboid joints. Figures 3A, 3B demonstrate the skin markings made preoperatively. Figure 3C shows the fibular bone graft with its associated distal vascular pedicle and Figure 3D demonstrates the position of the graft in the midfoot, marked by an arrow. Initially, a backslab was applied followed by a full fiberglass cast for two weeks. Follow-up CT and radiograph studies revealed excellent graft incorporation by nine months with no loosening or breakage of the metal plate. These are presented in Figure 4. The patient no longer required pain medication which consisted of opiates and gabapentin and at 10 months she had returned to light duties at work. When seen at a six-year follow-up, radiographs demonstrated good incorporation of the bone graft and no metal-ware failure, her visual analog score (VAS) being recorded as 1/10.

As this was an anonymous case report, patient consent was obtained in accordance with the principles of the Declaration of Helsinki.

## Discussion

Müller-Weiss disease (MWD)

To our knowledge, this is the first paper to describe the novel use of a reverse flow, vascularized fibular bone graft to treat recalcitrant MWD. Surgical procedures for MWD are mostly limited to joint fusion; this has variable success with considerable risks of pseudoarthrosis, as demonstrated in our case [7]. Levinson et al. described the use of a vascularized medial femoral condyle bone graft in a young male patient with MWD; this patient had a similar successful recovery [12].

Etiology

Historically, MWD was defined as spontaneous osteonecrosis of the navicular bone [1], this theory being disproven by Maceira and Rochera in 2004, where histopathological assessment demonstrated normal bone [5]. Current understanding suggests that any cause of prolonged lateral compression or uneven weight distribution to the navicular bone impairs its vascular supply and delays ossification [1]. The navicular is the final tarsal bone to ossify and in this weakened state, shearing forces across it may cause dorsolateral fragmentation [3,13]. Other possible causes of MWD include traumatic or biomechanical factors, perinavicular arthritis, and congenital malformation [3].

MWD is often compared with Kohler’s disease, an osteochondrosis of the navicular causing foot pain in children three to seven years old. This is a benign and self-limiting condition often without long-term sequelae and does not represent an early stage of MWD. Kohler’s disease usually occurs unilaterally, predominantly in young males whereas MWD typically occurs bilaterally in adult females [13].

Epidemiology

The incidence of MWD is unclear; in an epidemiological review of 191 patients with MWD, Maceira and Rochera identified a higher incidence of MWD in individuals who had experienced significant stressors such as war and extreme poverty, as well as trauma to the affected foot [5]. Hypotheses suggest an association between environmental and nutritional factors relating to these stressors and a generalized delay in development. This includes failure to reach growth potential, hypoplasia of the dental enamel, and importantly delayed ossification of the navicular bone [1]. However, Doyle et al. found no similar associations when reviewing presentations of MWD over a 10-year period [6].

Clinical Presentation

Patients with MWD typically present in the fifth or sixth decade with longstanding pain across the dorsal mid and hindfoot, often bilaterally as demonstrated in our case [1]. Examination may demonstrate swelling and tenderness in this region, with variable loss of the medial longitudinal arch [3]. A prominent navicular may cause the appearance of a heel valgus deformity; however, a varus deformity is often present on heel raise. This combination of pes planus and heel valgus is termed "pes planovarus," and this position forces the tibia into external rotation, altering the alignment of the knee and resulting in arthritic knee pain [1].

Imaging for MWD consists of weight-bearing radiographs of both feet and ankles to allow comparison with the normal anatomy in unilateral cases or progressive stages of disease in bilateral cases. Anteroposterior, lateral, and oblique views of the foot and lateral and mortise views of the ankle are desirable [13]. As well as establishing the degree of disease, weight-bearing radiographs may also exclude diagnoses of navicular stress fracture or arthritic changes due to trauma or rheumatoid arthritis [2]. Radiograph findings may include fragmentation of the navicular, osteophyte formation over the midtarsals, and widening of the talar head [3]. The navicular may appear hourglass-shaped on an AP film due to lateral compression and collapse [2]. Arthritic change and navicular collapse are identifiable in the initial X-rays of our patient in Figures 1, 2.

Classification

The severity of MWD may be classified into one of five stages, first described by Maceira and Rochera in 2004, and summarized in Table 1 [5]. These stages define the degree of deformity present on lateral weight-bearing radiographs using the Meary-Tomeno angle, which lies between the axes of the talus and the first metatarsal. Although this classification clearly describes the progression of MWD, it does not necessarily correlate with a patient’s symptoms or function [2]. However, no alternative has been suggested and this classification is still used in recent reviews of MWD management [7].

**Table 1:** Radiological staging of Müller-Weiss Disease Source: adapted from Maceira and Rochera [5]

Stage	Description
1	Normal radiographic appearance but may demonstrate subtalar varus deformity due to lateral displacement of the talar head which overlaps the anterior calcaneal process.
2	Dorsolateral subluxation of the talus causing dorsal angulation of the Meary-Tomeno line.
3	Compression or splitting of the navicular causing a lowered longitudinal arch and neutralization of the Meary-Tomeno line.
4	Compression of the navicular causing loss of the longitudinal arch and plantar angulation of the Meary-Tomeno line.
5	Articulation of the talus and cuneiform bones with medial and dorsal extrusion of the fragmented navicular.

Management

Patients with MWD may be offered a range of conservative and surgical options. Although there is no recommended treatment pathway for MWD, much of the published literature favors trialing conservative measures for at least six months before offering surgery [3,13]. Indications for surgery are based on symptom severity rather than physical deformity.

Conservative options consist of footwear and activity modification, analgesia, and physiotherapy. Orthotics should provide symptomatic relief by correcting the pes planovarus deformity associated with MWD and reinforcing the medial longitudinal arch. Activity modification may include reducing high-impact sports such as running to reduce midfoot strain and navicular stress. These measures including the trial of orthotics were insufficient to control our patient's symptoms and she therefore progressed to surgical intervention.

Where symptoms cannot be managed with conservative techniques alone, surgery may be an option. The aims of any operation for MWD are to stiffen the joints of the midfoot and restore the plantar arches to reduce the patient’s symptoms [2]. Surgical options described in the literature include internal fixation of the navicular, single or multiple joint fusions, and navicular excision and reconstruction of the medial tarsal column using a femoral head allograft [2,3,6,13]. The choice of operation may depend on the amount of healthy bone present and the degree of arthritic change. For example, internal fixation of the navicular may be used for acute fractures in the context of MWD, but this would not be possible in advanced disease due to the reduced amount of healthy bone stock. Possibilities for joint fusion may include talonavicular (TN), talonavicular-cuneiform (TN-C) or subtalar-talonavicular-calcaneocuboid (triple) fusions. TN and TN-C fusions offer good outcomes, using bone grafts, either autograft or allograft or metal screws to achieve fusion. Triple fusion may be beneficial if the subtalar or calcaneocuboid joints are involved; however, it does not address the degeneration between the navicular and cuneiform bones, potentially resulting in ongoing arthritic pain following surgery.

Fibular flap

Bone grafting is a useful method of bridging bony defects that may occur due to trauma, infection, malignancy, or congenital abnormalities [14]. Grafts may be classified as vascularized or non-vascularized. Bone healing in non-vascularized grafts is dependent on the vascularity of the surrounding tissues whereas vascularized grafts maintain an adequate blood supply, promoting bone remodeling and hypertrophy [14]. Vascularized grafts may be further classified into pedicled flaps, where the bone and overlying skin paddle are excised and rotated on the axis of the supplying artery to the position of the defect, or free flaps, where the osteocutaenous flap is fully excised and the supplying artery and vein are anastomosed to surrounding vessels at the site of the defect. Donor sites include the iliac crest, rib, radius, ulna, femur, humerus, pubis, and fibula. The FF describes removing a section of the fibula along with the overlying soft tissues and inserting it into the donor site, anastomosing the vasculature of the flap to the surrounding vessels. This technique was first officially described in 1975 by Taylor et al. for the surgical management of traumatic lower limb injuries that would have otherwise led to limb amputation [15]. Hidalgo later developed this procedure for mandibular reconstruction [16]; the osteocutaneous FF is now considered the gold standard in the reconstruction of the mandible following oncological resection [17]. The use of a FF in midfoot reconstruction for MWD is novel and not previously described.

Anatomy

The fibula receives a direct arterial supply from the peroneal artery, a branch of the posterior tibial artery. Branches of the peroneal artery are divided into major and minor pedicles. The major pedicle supplies the endosteum of the fibula via the nutrient foramen, located on the posterior middle third of the bone. The nutrient foramen should be included in the FF to ensure a successful vascular transfer. The minor pedicle forms a net-like structure around the periosteum, supplying the outer third of the fibula. The peroneal artery also supplies perforating branches to the overlying skin [14].

The reverse FF is based on retrograde flow in the peroneal vessels from distal anastomoses, and the bone flap is pedicled distally on these vessels and inserted into the midfoot as demonstrated in Figure 3.

Technique

Preoperative vascular mapping is recommended to identify vascular changes that may affect the viability of the flap and guide the techniques used in theatre [18]. This may include Doppler assessment, CT, or MRI angiography and aims to identify conditions such as peripheral vascular disease or anatomical variations such as a "peronea arteria magna," where one or both tibial arteries are absent. In these situations, harvesting the peroneal artery may rapidly lead to acute ischemia of the lower limb. There is debate in the literature whether a normal history and examination of the patient is sufficient to avoid the additional financial and time cost of vascular mapping [19]. Alolabi et al. demonstrated that in 132 patients assessed for an FF, the surgical plan was altered in 12.9% of patients based on MR angiography findings [20]. It is suggested that vascular mapping may increase the likelihood of success of the procedure by allowing proper consideration of any vascular changes present and the implications of these [19].

The fibula is accessed using a lateral approach; however, this may be modified when using osteocutaneous or osteomuscular flaps [14]. The peroneal artery and venae comitantes are identified as well as the location of the nutrient artery entering the fibula, this is most often in the middle third of the bone [14]. The length of the fibula excised is calculated by the size of the defect, this may measure up to 25 cm in length [20]. Ideally, at least 6 cm of proximal bone is left to avoid damaging the peroneal nerve and 4 cm of bone is left distally to maintain the stability of the ankle joint [14]. In the reverse flow FF, the excised fibula is pedicled on the distal vessels into the defect. As blood flow through the graft is now in an opposite direction to the rest of the limb, the graft is referred to as “reverse flow.”

Outcomes

Early complications of an FF may include wound infection and necrosis, partial or total loss of the skin graft, and uncontrolled bleeding. Donor site compartment syndrome may occur, and the graft may become necrotic if the vascular pedicle is distorted. Postoperatively, initial pain on weight-bearing would be expected; however, a return to a normal gait pattern is seen three months post-surgery [21]. Late complications may include a reduced range of movement and instability of the ankle joint. If the peroneal nerve has been damaged intraoperatively, the patient may notice weakened dorsiflexion of the great toe. Patients may experience moderate foot drop if the flexor hallucis longus muscle has been disrupted when raising the FF [21]. Belt et al. suggested a greater likelihood of stress fractures of the graft due to its reduced strength, describing full hypertrophy of the bone occurring at 18 months [22].

## Conclusions

The management of MWD is extremely difficult, and multiple procedures may be required to restore a pain-free gait. This case describes the successful use of a reverse vascularized fibular bone graft to facilitate a revision fusion of the midfoot. This procedure was successful, with a reduction in reported pain and good bone fusion demonstrated on serial imaging.

This is the first documented use of a reverse vascularized fibular bone graft for recalcitrant MWD. This graft is easily incorporated into the midfoot, relieving the severe symptoms of MWD described in this case. It should be considered to become an integral part of the surgical treatment of this painful and debilitating disease of the midfoot.
